# Characterization of the Deamination Coupled with Sliding along DNA of Anti-HIV Factor APOBEC3G on the Basis of the pH-Dependence of Deamination Revealed by Real-Time NMR Monitoring

**DOI:** 10.3389/fmicb.2016.00587

**Published:** 2016-04-28

**Authors:** Keisuke Kamba, Takashi Nagata, Masato Katahira

**Affiliations:** ^1^Institute of Advanced Energy, Kyoto UniversityKyoto, Japan; ^2^Graduate School of Energy Science, Kyoto UniversityKyoto, Japan

**Keywords:** APOBEC3G, NMR, monitoring, enzyme, HIV-1

## Abstract

Human APOBEC3G (A3G) is an antiviral factor that inactivates HIV. The C-terminal domain of A3G (A3G-CTD) deaminates cytosines into uracils within single-stranded DNA (ssDNA), which is reverse-transcribed from the viral RNA genome. The deaminase activity of A3G is highly sequence-specific; the third position (underlined) of a triplet cytosine (CCC) hotspot is converted into CCU. A3G deaminates a CCC that is located close to the 5′ end of ssDNA more effectively than ones that are less close to the 5′ end, so-called 3′ → 5′ polarity. We had developed an NMR method that can be used to analyze the deamination reaction in real-time. Using this method, we previously showed that 3′ → 5′ polarity can be explained rationally by A3G-CTD's nonspecific ssDNA-binding and sliding direction-dependent deamination activities. We then demonstrated that the phosphate backbone is important for A3G-CTD to slide on the ssDNA and to exert the 3′ → 5′ polarity, probably due to an electrostatic intermolecular interaction. In this study, we investigate the pH effects on the structure, deaminase activity, and 3′ → 5′ polarity of A3G-CTD. Firstly, A3G-CTD was shown to retain the native structure in the pH range of 4.0–10.5 by CD spectroscopy. Next, deamination assaying involving real-time NMR spectroscopy for 10-mer ssDNA containing a single CCC revealed that A3G-CTD's deaminase activity decreases as the pH increases in the range of pH 6.5–12.7. This is explained by destabilization of the complex between A3G-CTD and ssDNA due to the weakened electrostatic interaction with the increase in pH. Finally, deamination assaying for 38-mer ssDNA having two CCC hotspots connected by a long poly-adenine linker showed that A3G-CTD retains the same pH deaminase activity preference toward each CCC as that toward the CCC of the 10-mer DNA. Importantly, the 3′ → 5′ polarity turned out to increase as the pH decreases in the range of 6.5–8.0. This suggests that A3G-CTD tends to continue sliding without abortion at lower pH, while A3G-CTD tends to dissociate from ssDNA during sliding at higher pH due to the weakened electrostatic interaction.

## Introduction

Human APOBEC3G (A3G), which belongs to a protein family known as apolipoprotein B mRNA-editing enzyme catalytic polypeptides, exhibits cytidine deaminase activity and converts cytosines (Cs) in single-stranded DNA (ssDNA) into uracils (Us) (Harris and Liddament, 2004; Kitamura et al., 2011; Aydin et al., 2014). A3G targets the newly reverse-transcribed minus ssDNA of human immunodeficiency virus 1 (HIV-1) and converts C to U, which results in introduction of a significant amount of guanine (G) to adenine (A) mutations in the HIV-1 genome (Harris et al., 2003; Zhang et al., 2003; Aydin et al., 2014). Using this activity, A3G inactivates HIV-1 that is deficient of viral infectivity factor (Vif) (Sheehy et al., 2002; Harris et al., 2003; Lecossier et al., 2003; Zhang et al., 2003).

A3G comprises two zinc-coordinating domains: a catalytically inactive N-terminal domain (A3G-NTD) and an active C-terminal domain (A3G-CTD) (Harris and Liddament, 2004; Haché et al., 2005; Navarro et al., 2005; Kitamura et al., 2011; Aydin et al., 2014). A3G-NTD mediates encapsidation and RNA binding (Khan et al., 2005, 2007; Navarro et al., 2005). On the other hand, A3G-CTD is the domain responsible for C to U deamination within ssDNA, and thereby for antiviral capability (Navarro et al., 2005; Browne et al., 2009; Kobayashi et al., 2014). Both A3G-NTD and A3G-CTD possess Zn^2+^-coordinating motif, His-X-Glu-X_27−28_-Pro-Cys-X_2_-Cys, and this motif is required for A3G-CTD to exert the catalytic activity (Harris and Liddament, 2004). Zn^2+^ is coordinated by histidine, two cysteines, and a water molecule, and glutamate is thought to exchange hydrogens during catalysis (Barnes and Smith, 1993; Betts et al., 1994; Carter, 1995; Xiang et al., 1995). This nucleophilic hydroxyl is responsible for A3G-CTD's deamination activity (Barnes and Smith, 1993; Betts et al., 1994; Carter, 1995). The molecular structure of A3G-CTD in its free form was obtained previously by means of crystallography and NMR (Chen et al., 2008; Holden et al., 2008; Furukawa et al., 2009; Harjes et al., 2009; Shandilya et al., 2010; Lu et al., 2015). A3G-CTD has an αA-β1-β2/β2′-αB-β3-αC-β4-αD-β5-αE-αF fold, which forms a globular domain composed of five β-sheets, flanked by six α-helices. However, how A3G-CTD interacts with ssDNA is still not well understood.

**Figure 1:**
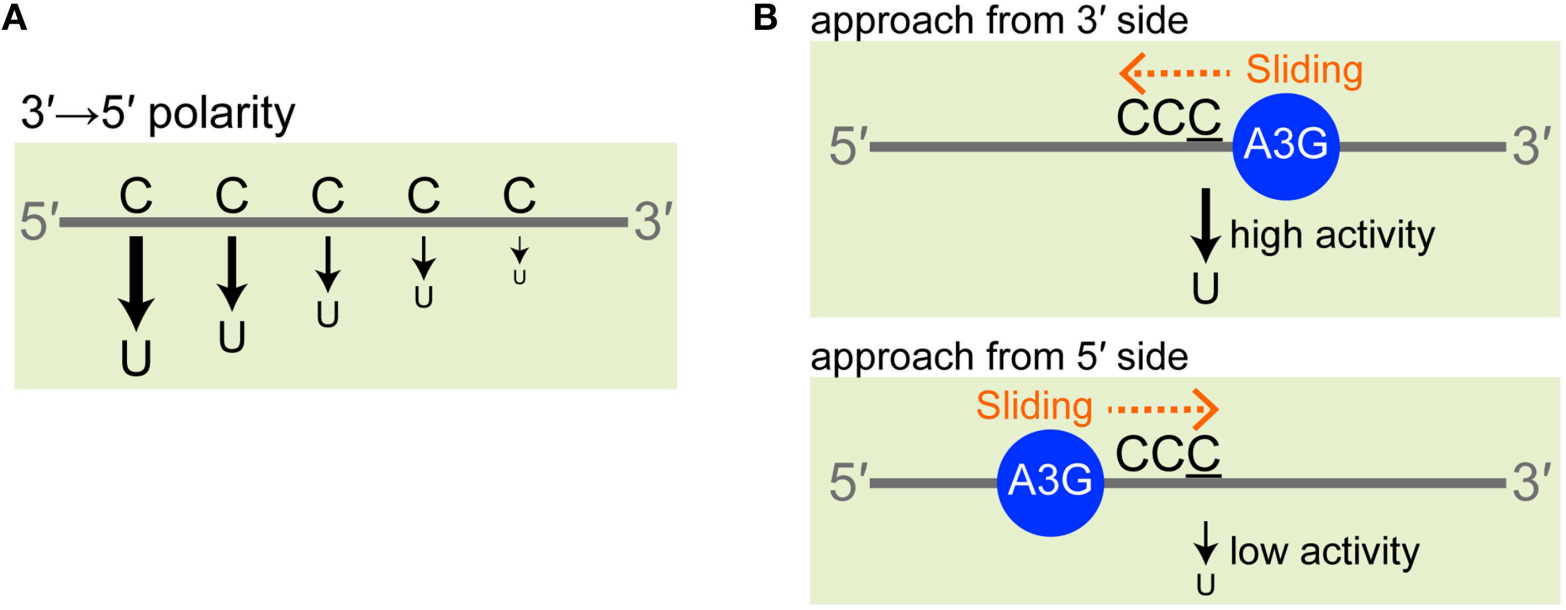
**3′ → 5′ polarity of A3G. (A)** A3G deaminates a CCC hotspot that is located close to the 5′ end more effectively than ones that are less close to the 5′ end. For simplicity, the third C of a CCC hotspot is indicated by a single “C” character. **(B)** The analysis of the data obtained by real-time NMR monitoring method revealed that deamination is more efficient when A3G-CTD approaches the target cytosine in the 3′ to 5′ direction rather than the opposite one and that this confers the 3′ → 5′ polarity.

The deaminase activity of A3G is ssDNA-specific and no double-stranded DNA or RNA with any structure can act as a substrate; it is also highly sequence-specific, i.e., a triplet cytosine (CCC) hotspot is converted into CCU, namely the third position of CCC is the most favored (Harris et al., 2003; Langlois et al., 2005; Chelico et al., 2006; Iwatani et al., 2006; Furukawa et al., 2009; Harjes et al., 2013; Kamba et al., 2015). Importantly, A3G exhibits a deamination bias, so-called 3′ → 5′ polarity. *In vitro*, A3G deaminates a CCC hotspot that is located close to the 5′ end more effectively than ones that are less close to the 5′ end (Chelico et al., 2006; Holden et al., 2008; Furukawa et al., 2014) (Figure 1A). This phenomena is called 3′ → 5′ polarity (Chelico et al., 2006), because the efficiency of deamination increases in the 3′ → 5′ direction. Just A3G-CTD alone reportedly exhibits this 3′ → 5′ polarity (Holden et al., 2008; Furukawa et al., 2014; Kamba et al., 2015). A3G's 3′ → 5′ polarity has long been observed also *in vivo* as a 5′ → 3′ gradient of G to A hyper-mutations in the HIV genome, which was replicated using minus ssDNA having C to U mutations introduced by A3G (Yu et al., 2004; Suspène et al., 2006; Kobayashi et al., 2014).

The deaminase activity of A3G has long been evaluated *in vitro* by means of various biochemical methods, i.e., uracil-DNA glycosylase-based assaying (Harris et al., 2003; Yu et al., 2004; Navarro et al., 2005; Chelico et al., 2006; Iwatani et al., 2006; Holden et al., 2008). In order to monitor the deamination reaction in real-time, and in a site-specific manner with high temporal and spatial resolution, we developed an NMR-based method (Furukawa et al., 2009, 2014; Kamba et al., 2015). Our real-time NMR monitoring method is increasingly being used to evaluate the deamination reaction of A3G (Harjes et al., 2013; Kouno et al., 2015; Lu et al., 2015), as well as that of other APOBEC family protein (Byeon et al., 2013). We also showed that this method can be applied to monitor the deamination of modified cytosines. We demonstrated that A3G-CTD possesses weak deaminase activity for 5-methyl cytosine (5mC), but none for 5-hydroxymethyl cytosine (5hmC), both of which are known to be epigenetic modifications (Kamba et al., 2015).

We have been using our real-time NMR monitoring method to explore the origin of the 3′ → 5′ polarity. By applying a kinetic model to the data obtained with this method, we showed that deamination is more efficient when A3G-CTD approaches the target cytosine in the 3′ to 5′ direction rather than the opposite one and that this sliding-direction-dependent deaminase activity confers the 3′ → 5′ polarity (Furukawa et al., 2014) (Figure 1B). We then used ssDNAs containing a nucleotide analog at a single or multiple positions as substrates for the real-time NMR monitoring method (Kamba et al., 2015). We demonstrated that five consecutive nucleotides, including CCC at the center, are recognized by A3G-CTD for efficient activity. Then, it was shown that the phosphate backbone is important for A3G-CTD to slide on the DNA strand and thereby to exert the 3′ → 5′ polarity (Kamba et al., 2015). Subsequent analysis to investigate the dependence of the 3′ → 5′ polarity on salt concentration suggested that the electrostatic intermolecular interaction tethers A3G-CTD to ssDNA, probably through interaction via the phosphate backbone (Kamba et al., 2015). In the present study, we investigate the pH effects on the structure, deaminase activity, and 3′ → 5′ polarity of A3G-CTD by using our real-time NMR monitoring method.

## Materials and methods

### Preparation of the protein and DNA substrates

Recombinant A3G-CTD (residues 193–384) containing an N-terminal hexahistidine tag (his-tag) was expressed in *Escherichia coli* BL21 (DE3/RIL) cells (Stratagene) and purified as described previously (Furukawa et al., 2009). Following cleavage of the his-tag and column purification, the obtained A3G-CTD was dialyzed against the storage buffer comprising 20 mM Tris-HCl (pH 7.5), 30 mM NaCl, 5 mM DTT, and 10 μM ZnCl_2_, and then stored until use at 4°C. All substrate ssDNAs (Table 1) were purchased from Fasmac Co., Ltd.

**Table 1:** **Substrate ssDNAs used in this study**.

**Substrate name**	**Sequence^a^**	**Length**	**Position of target C**
s10-1	ATTCCCGATT	10	6
s10-2	AAACCCGAAA	10	6
s38	AAACCCGA_24_CCCGTAA	38	6 and 34

a *The target cytosines deaminated by A3G are underlined*.

### Circular dichroism (CD) and fluorescence spectroscopy

Samples were prepared by mixing A3G-CTD in storage buffer with several measurement buffers having different pHs (1:19 ratio). The final concentration of A3G-CTD was adjusted to 6 μM. Different buffer systems were used to obtain different pH solutions: Glycine-HCl (pH 2.5–3.5), Acetic acid-Sodium acetate (pH 4.0–5.5), Bis-Tris-HCl (pH 6.0–7.0), Tris-HCl (pH 7.5–8.5), CHES-NaOH (pH 9.0–9.5), CHAPS-NaOH (pH 10.0–11.0), and Triethylamine-HCl (pH 11.4–12.7). The concentrations of these buffers were adjusted to 20 mM, and 30 mM NaCl, 1 mM DTT and 10 μM ZnCl_2_ were added to obtain the measurement buffers. Equilibrium denaturation was carried out using different concentrations of guanidine hydrochloride (GdHCl) in the range of 0 to 5.8 M at pH 7.5. All CD and fluorescence measurements were carried out at 25°C.

CD spectra in the far-UV region (200–260 nm) using an optical path cell of 0.1 cm were obtained with a Jasco J-720A spectrometer (Japan Spectroscopic Co.). All spectra were background-corrected, smoothed, and converted to molar ellipticity [θ] (deg·cm^2^·dmol^−1^) (Kelly et al., 2005).

Fluorescence spectra were collected with a Jasco FP-8500 spectrometer (Japan Spectroscopic Co.) using an optical path cell of 1 cm. The excitation wavelength was 295 nm and the fluorescence intensity at the emission wavelength of 335 nm was monitored. The spectra slit width of 5 nm was used for excitation and emission.

The thermodynamic parameters for guanidine-induced denaturation curves were obtained by least squares fitting using two-state equations (Pace, 1986; Scholtz et al., 2009).

(1) $$\begin{array}{r} s_{\text{obs}}=\frac{\begin{array}{c} (a_{\text{N}}\;+\;b_{\text{N}}[\text{GdHCl}])+(a_{\text{U}}+b_{\text{U}}[\text{GdHCl}])\exp\left(\frac{-\Delta G_{\text{app}}+m_{\text{app}}[\text{GdHCl}]}{RT}\right) \end{array}}{1+\;\exp\left(\frac{-\Delta G_{\text{app}}+m_{\text{app}}[\text{GdHCl}]}{RT}\right)} \end{array}$$

The data were fitted using Equation (1), where *s*_obs_ is the molar ellipticity or fluorescence intensity observed; *a*_N_ (*a*_U_) and *b*_N_ (*b*_U_) the intercept and slope of the base line at low (N) and high (U) GdHCl concentrations, respectively; *R* the gas constant; *T* the absolute temperature in K; [GdHCl] the GdHCl concentration; and Δ*G*_app_ and *m*_app_ the free energy and cooperativity of folding calculated with Equation (1), respectively. The errors were estimated using a single-elimination Jack-knife procedure (Harris, 1998).

The fraction of the folded state (*f*_N_) at each pH was calculated using the equation,

(2) $$\begin{array}{l} f_{\text{N}}\;=\left(\text{Y}-{\text{Y}}_{\text{U}}\right)/\left({\text{Y}}_{\text{N}}-{\text{Y}}_{\text{U}}\right) \end{array}$$

where Y is the [θ] value at 220 nm ([θ]_220_) at each pH. Y_N_ and Y_U_, corresponding to *a*_N_ and *a*_U_ indicated above, respectively, are [θ]_220_ for the folded and unfolded states at pH 7.5 and [GdHCl] = 0, respectively (Y_N_ = −5.2 × 10^3^, Y_U_ = −2.5 × 10^3^) (Scholtz et al., 2009).

### NMR spectroscopy

NMR spectra were recorded at 25°C with either a DRX600 or Avance III 600 spectrometer, both being equipped with a cryogenic probe and a Z-gradient (Bruker Biospin).

Samples were prepared by mixing A3G-CTD in storage buffer with several measurement buffers having different pH. The measurement buffers were prepared as in the previous *s*ection, except that DTT was adjusted to 5 mM. The concentration of A3G-CTD was set either at 0.8 or 2.0 μM, while that of the ssDNA substrates was adjusted to 200 μM. After mixing A3G-CTD and ssDNA together, a series of two-dimensional TOtal Correlated SpectroscopY (2D TOCSY) with mixing a time of 20 ms were recorded at different reaction time points, by which the deamination reaction of cytosines of ssDNA could be monitored in real-time. Water signal suppression was achieved with 3-9-19 watergate. Resonance assignments of the substrate ssDNAs were made using 2D TOCSY with an aid of 2D ^1^H–^13^C Heteronuclear Single Quantum Coherence (HSQC) spectroscopy and also by comparing the chemical shifts with the assignments for ATTCCCAATT, AAACCCGAAA, and AAACCCGA_24_CCCGTAA, previously studied (Furukawa et al., 2009; Kamba et al., 2015). Spectra were processed with NMRPipe (Delaglio et al., 1995), and analyzed using SPARKY (Goddard and Kneller, 2006).

The intensities of the peaks of either cytosine or uracil were plotted against time and analyzed as previously described (Furukawa et al., 2014; Kamba et al., 2015). The data were fit to a single exponential decaying function, using the following equations, by which apparent deamination rate constants were derived:

(3) $$\begin{array}{l} {I\left(t\right)}_{\text{C}}=I_{\text{s}}\;\exp\left(-\;kt\right)\;+\;I_{\text{b}} \end{array}$$

(4) $$\begin{array}{l} {I\left(t\right)}_{\text{U}}\;=\;I_{\text{s}}\left(1\;-\exp\left(-kt\right)\;\right)\;+I_{\text{b}} \end{array}$$

where *I*_b_ corresponds to the baseline intensity of the spectrum; *I*_s_ + *I*_b_ the signal intensity at time point zero for cytosine (3) and ∞ for uracil (4); and *k* the apparent deamination rate constant, which was always obtained through linear least-squares analysis. The value for the apparent deamination rate constant was used as an index of activity.

We defined the *P* with the following equation:

(5) $$\begin{array}{r} P=\frac{k_{\left(\text{C}6\right)}}{k_{\left(\text{C}34\right)}} \end{array}$$

where *k*_(*C*6)_ and *k*_(*C*34)_ are the apparent deamination rate constants at positions C6 and C34 of a 38-mer ssDNA (Table 1, s38). “*P*” can be regarded as a measure of the magnitude of 3′ → 5′ polarity. The error was estimated as follows. Firstly, the error of the NMR signal intensity was obtained from the base level noise of each NMR spectrum. Then the error of the rate constant was calculated from data sets constructed by Monte Carlo simulation using the error of the NMR signal intensity. Finally, the errors of the relative activity and the *P* were obtained by means of error propagation calculation.

## Results and discussion

### Real-time NMR monitoring of cytosine deamination catalyzed by A3G-CTD

**Figure 2:**
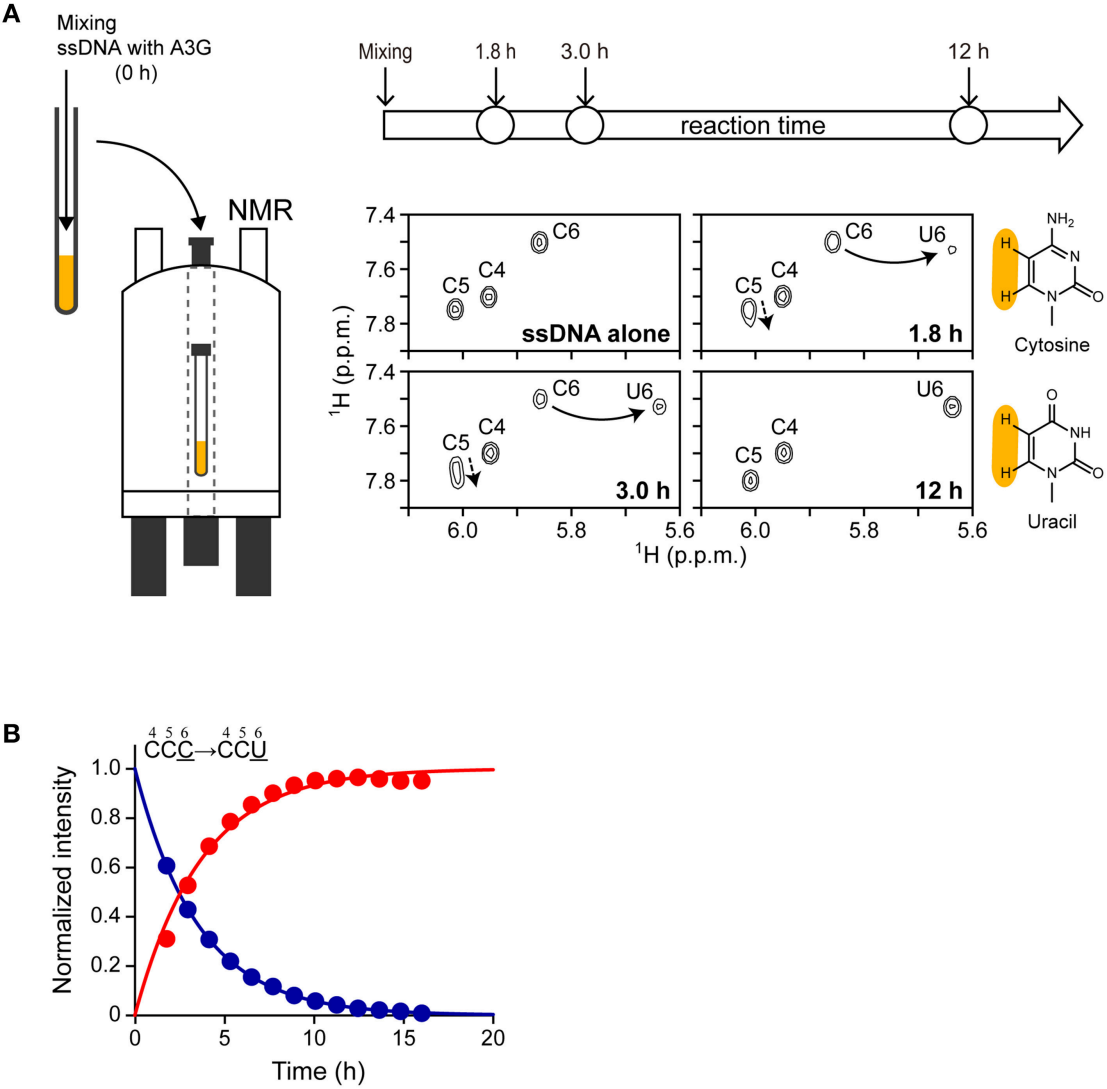
**Overview of real-time NMR monitoring**. (**A**, left) Illustration of the experimental procedure. The solutions containing A3G-CTD and ssDNA substrate were mixed together (reaction time point 0 h), and the mixture was transferred to an NMR tube. The NMR tube was then loaded into the magnet. A series of 2D TOCSY spectra was recorded at different reaction time points. (**A**, right) Here, a reaction for ATTCCCGATT (s10-1) is monitored as an example. C to U conversion occurred at the underscored cytosine (C6). The panels show the regions of 2D TOCSY spectra for H5–H6 correlation signals of cytosines and uracils at different reaction time points (1.8, 3.0, and 12 h). The spectrum of “ssDNA (s10-1) alone” is shown for comparison. The structural formulas of cytosine and uracil are shown (protons H5 and H6 are highlighted in yellow). Signals for C6 are connected with those for U6 by solid arrows. Signals for C4 and C5 are perturbed (broken arrows) due to the C6 to U6 conversion. **(B)** The intensities of H5–H6 correlation signals for C6 and U6 at different reaction time points are plotted. The data were fitted to a single exponential function, by which the rate constant, *k*, was obtained.

We have been utilizing our real-time NMR monitoring method to investigate the deamination reaction of A3G and A3G-CTD (Furukawa et al., 2009, 2014; Kamba et al., 2015). The experimental procedure for the method is illustrated in Figure 2A (left). We add A3G-CTD to the solution containing ssDNA and mix thoroughly, which is defined as reaction time point zero. The final concentrations of A3G-CTD and s10-1 are 2 and 200 μM, respectively. The mixed solution is immediately transferred to an NMR tube, which is loaded into the magnet. Subsequently, a series of 2D TOCSY spectra are recorded at different reaction time points. The conversion of ATTCCCGATT (s10-1 in Table 1) to ATTCCUGATT is monitored as an example in Figure 2A (right). The panels in Figure 2A (right) shows regions of 2D TOCSY spectra for H5–H6 correlation signals of cytosines and uracils. The three signals in the top-left panel correspond to the C4, C5, and C6 residues of s10-1. With the reaction time, the intensity of the C6 signal gradually decreases and it eventually disappears, which is coupled with the emergence of the U6 signal and a gradual increase of its intensity (Figure 2A, panels 1.8, 3.0, and 12 h). We then collect the intensities of these signals and follow the time courses of their changes. In the current case, the signal intensity at each time point is plotted in Figure 2B. The plots for C6 and U6 were each fitted to a single exponential function, Equations (3, 4) in the Materials and Methods section, by which the rate constant, *k*, was found to be 0.28 ± 0.01 and 0.27 ± 0.02 h^−1^, respectively. This *k*-value can be used as an index of activity.

### The effects of pH and denaturant on the structure of A3G-CTD

**Figure 3:**
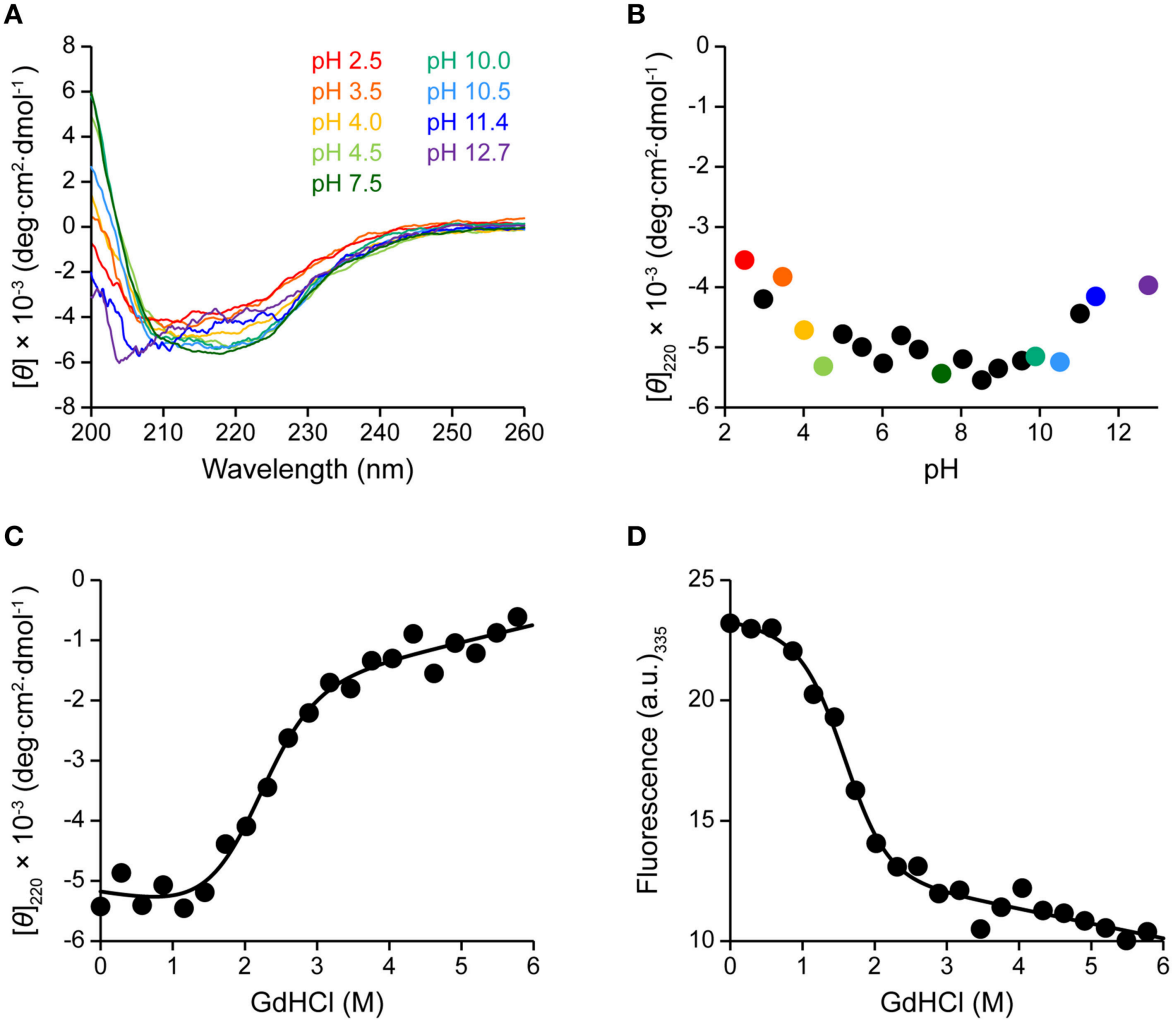
**Effects of pH and denaturant on the structure of A3G-CTD as assessed by CD and fluorescence spectroscopy. (A)** Far-UV CD spectra of A3G-CTD at pH 2.5, 3.5, 4.0, 4.5, 7.5, 10.0, 10.5, 11.4, and 12.7 are shown. **(B)** Molar ellipticity at 220 nm of A3G-CTD is plotted against pH. The color-coded points corresponds to the corresponding spectra in **(A)**. **(C,D)** Guanidine-induced denaturation of A3G-CTD was investigated at pH 7.5. The molar ellipticity at 220 nm **(C)** and fluorescence intensity at 335 nm **(D)** were each plotted against the concentration of guanidine hydrochloride (GdHCl).

Here we assessed the structure of A3G-CTD in buffers having different pHs using CD spectroscopy. Far-UV (200–260 nm) CD spectra were obtained at nine different pH values within the range of 2.5–12.7 (pH 2.5, 3.5, 4.0, 4.5, 7.5, 10.0, 10.5, 11.4, and 12.7; Figure 3A). The patterns of the spectra in the range of pH 4.0–10.5 were almost identical to that of the spectrum at pH 7.5, where A3G-CTD reportedly exerts deaminase activity (Holden et al., 2008; Harjes et al., 2013; Furukawa et al., 2014; Kamba et al., 2015), indicating that A3G-CTD is in the native form within this pH range. Indeed, the spectra in the range of pH 4.0–10.5 show strong negative intensity around the wavelength range of 210–230 nm, which is generally known as an indication of the presence of secondary structures (Kelly et al., 2005). In Figure 3B, the variation in the molar ellipticity at 220 nm ([θ]_220_) of A3G-CTD at 20 different pH values in the range of 2.5–12.7 is showed. The average value of [θ]_220_ was −5.1 × 10^3^ deg·cm^2^·dmol^−1^ in the range of pH 4.0–10.5, where A3G-CTD has the native structure, as mentioned above. However, at pH values lower and higher than pH 4.0 and 10.5, respectively, the values of [θ]_220_ were higher (ca. −4.0 × 10^3^ deg·cm^2^·dmol^−1^), which implies that the content of the secondary structures was somehow lower.

Next, to evaluate whether or not A3G-CTD is in the native form in the region of pH < 4.0 and pH > 10.5 ([θ]_220_ value of ca. −4.0 × 10^−3^), we conducted guanidine-induced denaturation of A3G-CTD at pH 7.5. The [θ]_220_ value was plotted against the concentration of GdHCl (Figure 3C). Then, we fitted the plotted data to Equation (1) in the Materials and Methods section to obtain a guanidine-induced denaturation curve, which describes a two-state transition model, and determined the apparent unfolding free energy (Δ*G*_app_) and cooperativity (*m*_app_) values to be 3.3 ± 0.3 kcal·mol^−1^ and 1.6 ± 0.1 kcal·mol^−1^·M^−1^, respectively (Table 2). For reference, we also investigated guanidine-induced denaturation of A3G-CTD at pH 7.5 using fluorescence spectroscopy (Figure 3D). The fluorescence spectrum with zero GdHCl showed a maximum at 335 nm, while at the highest GdHCl concentration (5.8 M), the maximum was at 348 nm (red-shifted), which strongly suggests that the tertiary structure of A3G-CTD has denatured almost completely (Royer, 2006). Here, fluorescence intensity at 335 nm was plotted against the concentration of GdHCl and fitted to the aforementioned Equation (1), and the Δ*G*_app_ and *m*_app_ values were determined to be 3.2 ± 0.4 kcal·mol^−1^ and 2.0 ± 0.2 kcal·mol^−1^·M^−1^, respectively (Table 2). The [θ]_220_ value of the CD spectrum and the fluorescence intensity at 335 nm are generally appreciated to be probes to determine the contents of secondary and tertiary structures, respectively (Royer, 2006). Since, the Δ*G*_app_ values obtained on CD and fluorescence spectroscopies are the same within error, the unfolding of A3G-CTD's secondary and tertiary structures is highly cooperative.

**Table 2:** **Thermodynamic parameters for guanidine-induced denaturation of A3G-CTD obtained by CD and fluorescence spectroscopy**.

**CD**		**Fluorescence**	
**Δ*G*_*app*_ (kcal·mol^−1^)**	***m*_*app*_ (kcal·mol^−1^·M^−1^)**	**Δ*G*_*app*_ (kcal·mol^−1^)**	***m*_*app*_ (kcal·mol^−1^·M^−1^)**
3.3 ± 0.3	1.6 ± 0.1	3.2 ± 0.4	2.0 ± 0.2

We then estimated the fraction of the folded state for A3G-CTD at different pH values using Equation (2). As a result, in the range of pH 4.0–10.5, the fraction of the folded state was found to be 90%, while it was 58% in the range of pH 11.0–12.7.

### The effect of pH on the deaminase activity of A3G-CTD

**Figure 4:**
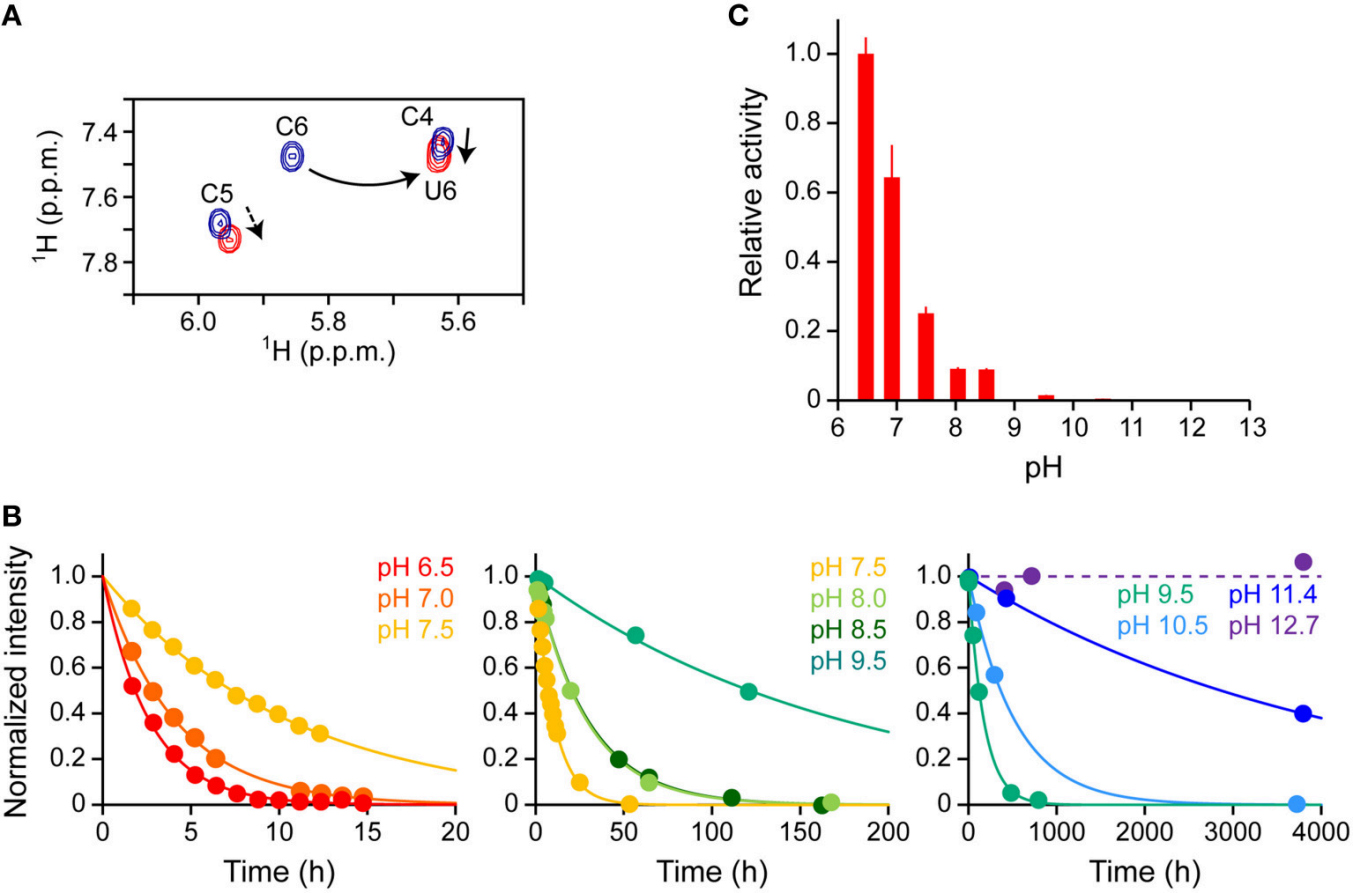
**Effect of pH on the deaminase activity of A3G-CTD for a 10-mer ssDNA as assessed by real-time NMR monitoring. (A)** Superpositioning of 2D TOCSY spectra of AAACCCGAAA (s10-2 in Table 1) at two reaction time points at pH 7.5 is presented as a representative. C to U conversion occurred at the underscored cytosine (C6). The H5–H6 correlation signals of cytosines and uracils are shown: blue and red signals correspond to reaction time points 1.6 and 53 h after addition of A3G-CTD, respectively. The signal of C6 is connected with that of U6 by a solid arrow. The signals of C4 and C5 are perturbed (broken arrows) due to the C6 to U6 conversion. **(B)** The time courses of the intensity changes were plotted for C6 of s10-2 at pH 6.5, 7.0, 7.5, 8.0, 8.5, 9.5, 10.5, 11.4, and 12.7. The data for each pH were fitted to a single exponential function individually, by which the rate constant, *k*, at each pH was obtained. **(C)** Relative activities for C6 are plotted against pH values. The relative activity at each pH was defined as the ratio of *k* at that particular pH to that obtained at pH 6.5.

Our real-time NMR monitoring method was applied to investigate the deaminase activity of A3G-CTD for a 10-mer ssDNA (s10-2), AAACCCGAAA (C to U conversion occurs at underscored cytosine, C6), at nine different pH values (pH 6.5, 7.0, 7.5, 8.0, 8.5, 9.5, 10.5, 11.4, and 12.7). In each experiment, the final concentrations of A3G-CTD and s10-2 were adjusted to 0.8 and 200 μM, respectively. A series of 2D TOCSY spectra for s10-2 were obtained at several reaction time points at each pH value. As an example, Figure 4A shows the spectra of s10-2 at pH 7.5, at time points 1.6 and 53 h after addition of A3G-CTD to s10-2 (blue and red signals, respectively). In Figure 4A, the H5–H6 correlation signals of C4 and U6 overlap, so the assignments were made with the aid of 2D ^1^H–^13^C HSQC spectroscopy to discriminate between C and U (data not shown).

We collected the intensities of C6 signals, which were isolated from other signals, and followed their decreases during the reaction time course for each pH value. The time courses of the intensity decrease are plotted in Figure 4B. The plot for each pH was fit to aforementioned single exponential decaying function, Equation (3), individually, and the rate constant, *k*, at each pH was obtained (Table 3). Since the *k*-value can be considered as an index of activity, we defined the relative activity as the ratio of *k* at a particular pH to that at pH 6.5. In the pH range studied here, the relative activity was highest at pH 6.5 and decreased as the pH increased (Figure 4C). It seems physiologically relevant that A3G-CTD exerts the highest activity around at the physiological pH, 6.5.

**Table 3:** **Relative activities of A3G-CTD for s10-2 at pHs ranging from 6.5 to 12.7**.

**pH**	**Relative activity (C6)**
6.5	1.00 ± 0.05
7.0	0.64 ± 0.10
7.5	0.25 ± 0.02
8.0	0.091 ± 0.005
8.5	0.089 ± 0.004
9.5	0.015 ± 0.001
10.5	(5.0 ± 0.3) × 10^−3^
11.4	(6.4 ± 0.5) × 10^−4^
12.7	n.d.

*n.d., not detected*.

The deaminase activity of A3G-CTD turned out to be higher at lower pH and decreased as the pH increased within the range of pH 6.5–11.4. ssDNA is negatively charged because it has phosphate moieties, therefore we suppose that this trend is due to a reduction of the stability of the complex between A3G-CTD and ssDNA due to the weakened electrostatic interaction with an increase in pH value. We also noticed that the activity of A3G-CTD was significantly decreased above pH 9.5 and become negligible around pH 12.7. According to the analysis in the previous section, the fraction of the folded state of A3G-CTD in the range of pH 9.5–12.7 was between 92 (pH 9.5) and 50% (pH 12.7). Therefore, we assume that the low deaminase activity in this pH range is due not only to the denaturation of A3G-CTD, but also to decreased stability of the complex between A3G-CTD and ssDNA. The decrease of deaminase activity of A3G-CTD spans in wide range (pH 6.5–11.4). This aspect seems not to be attributable to a single amino acid residue, but rather multiple amino acid residues seem to be involved. It may be possible to find the residues that cause pH dependency if many A3G-CTDs containing different mutations at multiple sites are used for analysis, but this is practically difficult.

We also applied the current analysis to the lower pH rang of pH 2.5–6.0. Within this pH range, the time course of the intensity decrease of C6 signals did not show simple single exponential decay, but rather a sigmoidal-like curve. The reasons for this phenomenon are not apparent, however, the protonation/deprotonation equilibria of histidine and/or cytosine may play roles. Previously, it was shown that the deaminase activity of A3G-CTD becomes maximum at pH 5.5 (Harjes et al., 2013). Since this pH is close to the pKa value of the imidazole of histidine (ca. 6.0 in general), it was suggested that the protonation of histidine 216 is the key to the promotion of the deaminase activity (Harjes et al., 2013). As for cytosine, we observed that the chemical shift values of the H5–H6 correlations were significantly perturbed at different pH values within the range of 2.5–6.0, which is supposed to be related to the protonation of cytosine. Our intention was to investigate the pH dependence of the 3′ → 5′ polarity of the deaminase activity, therefore we decided to carry out the subsequent experiments in the pH range of 6.5–12.7 to make the analysis simple.

### The effect of pH on the 3′ → 5′ polarity of A3G-CTD

**Figure 5:**
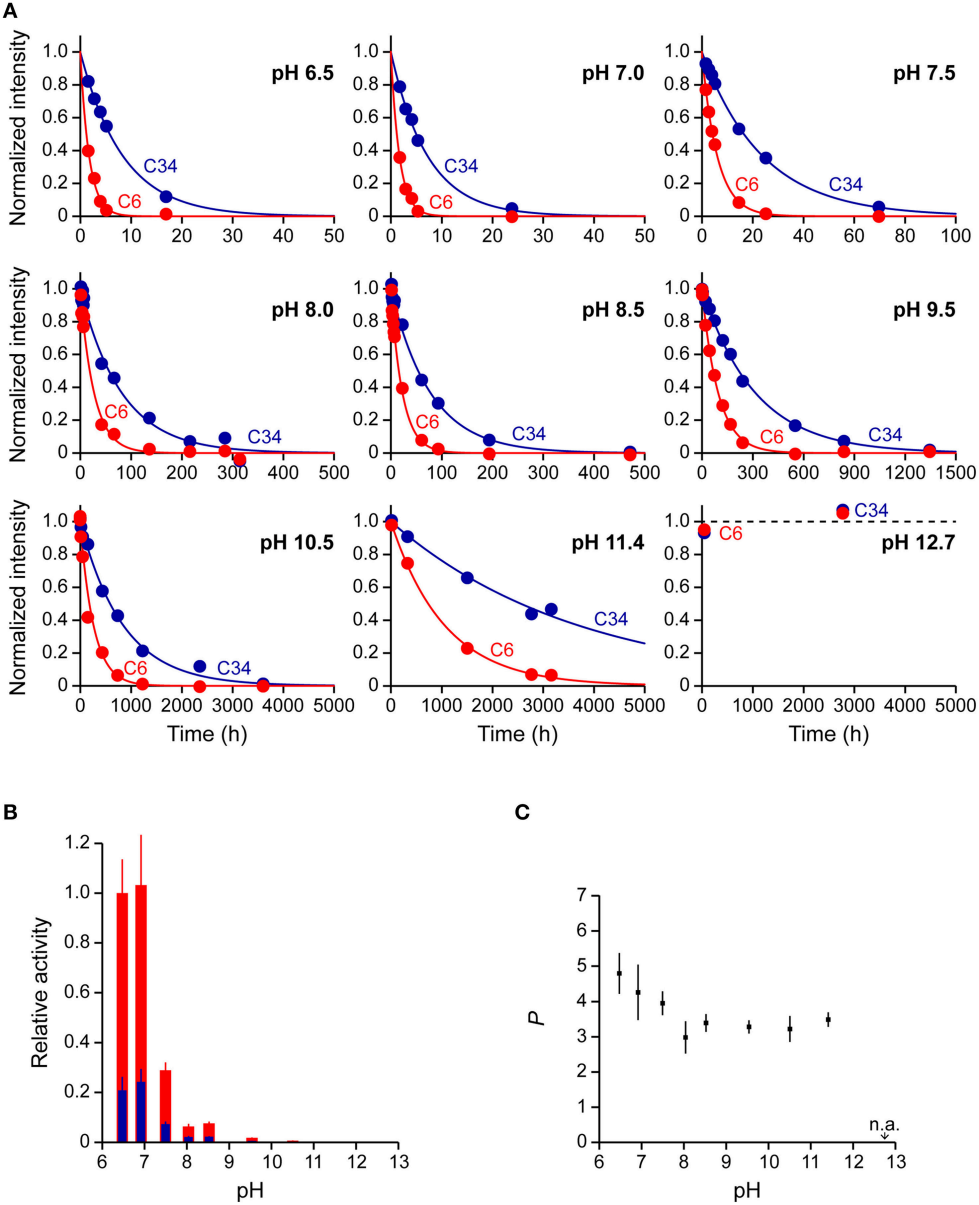
**Real-time NMR monitoring of deamination reactions for ssDNA having two CCC hotspots at various pHs. (A)** The time courses of the intensity changes were plotted for C6 and C34 of AAACCCGA_24_CCCGTAA (s38 in Table 1) at pH 6.5, 7.0, 7.5, 8.0, 8.5, 9.5, 10.5, 11.4, and 12.7. C6 and C34, which are converted to U, are underscored in the sequence. The data of C6 and C34 for each pH were fitted to a single exponential function individually, by which the rate constant, *k*, at each pH was obtained. **(B)** Relative activities for C6 (red) and C34 (blue) are plotted against pH values. The relative activities for C6 and C34 at each pH were defined as the ratio of their *k*s at each pH to that obtained for C6 at pH 6.5. **(C)** 3′ → 5′ polarity values (*P*) are plotted against pH values. The *P* at each pH is defined as the ratio of *k* obtained for C6 to that obtained for C34 (Equation 5).

We, next, used a 38-mer ssDNA substrate (s38), AAACCCGA_24_CCCGTAA, to investigate the deamination reaction of A3G-CTD at nine different pH values (pH 6.5, 7.0, 7.5, 8.0, 8.5, 9.5, 10.5, 11.4, and 12.7). This s38 contains two CCC hotspots, whose third positions, C6 and C34 (underlined in the sequence), are converted to uracil by A3G-CTD. In each experiment, the final concentrations of A3G-CTD and s38 were adjusted to 0.8 and 200 μM, respectively. A series of 2D TOCSY spectra for s38 at several reaction time points were obtained at each pH value in the same way as for s10-1 and s10-2 in the previous sections. We collected the intensities of C6 and C34 signals, and plotted them against time (Figure 5A). The plots for the C6 and C34 signals at each pH were each fitted to Equation (3) individually, by which the rate constants were obtained. The relative activities at different pHs were then calculated (Table 4), and are compared in Figure 5B. Here the relative activities for C6 and C34 at each pH were defined as the ratios of their *k*-values to the *k*-value obtained for C6 at pH 6.5. The pH dependence trends of the relative activity for C6 and C34 in s38 were the same in each case as the trend observed for C6 in s10-2; the *k*-values being highest at around pH 6.5–7.0 and decreased as the pH increased (Figure 5B). Again, the activity of A3G-CTD decreased significantly above pH 9.5 and became negligible around pH 12.7. At all pH values, the relative activity for C6 was higher than that for C34.

**Table 4:** **Relative activity and 3′ → 5′ polarity values (*P*) of A3G-CTD for s38 at pHs ranging from 6.5 to 12.7**.

**pH**	**Relative activity (C6)**	**Relative activity (C34)**	**3′ → 5′ polarity (*P*)**
6.5	1.00 ± 0.14	0.21 ± 0.06	4.8 ± 0.6
7.0	1.03 ± 0.21	0.24 ± 0.06	4.3 ± 0.8
7.5	0.29 ± 0.04	0.073 ± 0.011	3.9 ± 0.4
8.0	0.063 ± 0.011	0.021 ± 0.004	3.0 ± 0.5
8.5	0.076 ± 0.008	0.022 ± 0.002	3.4 ± 0.3
9.5	0.018 ± 0.002	(5.6 ± 0.4) × 10^−3^	3.3 ± 0.2
10.5	(6.6 ± 0.9) × 10^−3^	(2.1 ± 0.2) × 10^−3^	3.2 ± 0.4
11.4	(1.6 ± 0.2) × 10^−3^	(4.7 ± 1.1) × 10^−4^	3.5 ± 0.3
12.7	n.d.	n.d.	n.a.

*n.d., not detected; n.a., not applicable*.

Subsequently, the 3′ → 5′ polarity of A3G-CTD's deaminase activity for s38 at each pH value was quantified as the ratio of the rate constant obtained for C6 to that obtained for C34 (Figure 5C and Table 4). *P* showed the highest value of ca. 4.8 at pH 6.5 and decreased as the pH increased, and then *P* reached a plateau with a value of ca. 3.5 at pH 8.0 and stayed almost the same to pH 11.4.

Previously, we showed that A3G-CTD does not exert 3′ → 5′ polarity for an ssDNA substrate whose two CCC hotspots are linked by a double-stranded DNA stretch (Furukawa et al., 2014). Additionally, we reported that the single-stranded nucleotide linker does not have to be DNA, but it can be either RNA or abasic DNA for A3G-CTD to exert the 3′ → 5′ polarity (Kamba et al., 2015). Then, we revealed that the electrostatic intermolecular interaction between A3G-CTD and ssDNA is the key for sliding, by demonstrating that the lower the NaCl concentration the higher the 3′ → 5′ polarity (Kamba et al., 2015). These findings indicated that the 3′ → 5′ polarity occurs because A3G-CTD slides along the phosphate backbone of the ssDNA stretch between the two CCC hotspots (Kamba et al., 2015). In the current study, we investigated the pH dependence of the 3′ → 5′ polarity. The results indicated that the lower the pH, the higher the 3′ → 5′ polarity in the range of pH 6.5–8.0. This can be explained by that the electrostatic intermolecular interaction between A3G-CTD and ssDNA is strengthened at lower pH, thereby the abortion of sliding is repressed, resulting in the high 3′ → 5′ polarity. Repression of the abortion of sliding enhances the deamination at the second hot spot after the deamination at the first hot spot. It is physiologically relevant that the repression of the abortion of sliding and thus the 3′ → 5′ polarity are highest around at the physiological pH, 6.5.

### Further improvement of temporal and spatial resolution of real-time NMR monitoring

In the present study, we obtained a series of 2D TOCSY spectra during the deamination reaction. Because it took about 75 min to obtain a single 2D TOCSY spectrum at each reaction time point, the temporal resolution was about 75 min. To achieve higher temporal resolution, one can introduce ^13^C-labeled cytosines to the substrate ssDNA and obtain a series of 2D ^1^H–^13^C HSQC spectra. The deamination can be monitored by means of 2D ^1^H–^13^C HSQC spectra through the gradual decrease and eventual disappearance of the H5–C5 correlation peak of a cytosine, which is coupled with the emergence and gradual increase of the H5–C5 correlation peak of a uracil. The measurement of ^1^H–^13^C HSQC spectra makes use of much larger one-bond ^1^H–^13^C coupling (${}^{1}J_{\text{HC}}$ = ~200 Hz) than the three-bond ^1^H–^1^H coupling (${}^{3}J_{\text{HH}}$ = ~7 Hz) used for the measurement of TOCSY spectra (Wijmenga and van Buuren, 1998; Fiala et al., 2004). Therefore, it is expected that the signal-to-noise ratio of a ^1^H–^13^C HSQC spectrum is much higher than that of a TOCSY spectrum, and that thus a ^1^H–^13^C HSQC spectrum can be obtained in a much shorter time period than a TOCSY spectrum. It might even be possible to monitor deamination using a 1D version of a 2D ^1^H–^13^C HSQC spectrum. If a 1D version of a ^1^H–^13^C HSQC spectrum can be obtained with a reasonable signal-to-noise ratio in one scan, the measurement time would be just 100 ms. Thus, with the proper relaxation delay for the next measurement, the temporal resolution of 300–1000 ms is supposed to be achieved in an ideal case.

The spatial resolution depends on the length of the ssDNA, and the numbers of cytosines and uracils contained in the sequence. So far, we have analyzed non-labeled ssDNA of as long as a 60-mer using 2D TOCSY spectra (Furukawa et al., 2014; Kamba et al., 2015). Residue-specific ^13^C-labeling of a cytosine of the substrate ssDNA can drastically increase the spatial resolution. It is supposed that ssDNA of a 100- to 300-mer can be analyzed by real-time NMR using 2D ^1^H–^13^C HSQC spectra in combination with specific ^13^C labeling.

Such improvement of the temporal and spatial resolution of real-time NMR monitoring will make this method even more valuable for investigating the characters and behaviors of various biological systems including enzymes.

## Conclusion

Here we presented methodology for the real-time NMR spectroscopy in detail, and described the ease of its usage, and the applicability to systems exhibiting different temporal and spatial resolution. This real-time NMR monitoring method was used to investigate the effects of pH on the deamination reaction and 3′ → 5′ polarity of A3G-CTD. A3G-CTD was firstly shown to retain its native structure fully or to some extent at the pH values studied (pH 4.0–12.7) by CD spectroscopy. Then real-time NMR spectroscopy was applied to a 10-mer ssDNA containing a single CCC, by which it was shown that the deaminase activity of A3G-CTD decreases as the pH increases. This was explained by a reduction in the stability of the complex between A3G-CTD and ssDNA due to the weakened electrostatic interaction with an increase in the pH value. Lastly, a 38-mer ssDNA having two CCC hotspots connected by a long poly-adenine linker was used to show that the 3′ → 5′ polarity of A3G-CTD increases as the pH decreases in the range of 6.5–8.0. This finding can be rationalized that stronger electrostatic intermolecular interaction between A3G-CTD and ssDNA at lower pH represses the abortion of sliding, resulting in high 3′ → 5′ polarity.

## Author contributions

Conceived and designed the experiments: KK, TN, MK. Performed the experiments: KK. Analyzed the data: KK, TN, MK. Contributed reagents/materials/analysis tools: TN, MK. Wrote the paper: KK, TN, MK.

### Conflict of interest statement

The authors declare that the research was conducted in the absence of any commercial or financial relationships that could be construed as a potential conflict of interest.
